# Dynamics of Archaeal and Bacterial Communities in Response to Variations of Hydraulic Retention Time in an Integrated Anaerobic Fluidized-Bed Membrane Bioreactor Treating Benzothiazole Wastewater

**DOI:** 10.1155/2018/9210534

**Published:** 2018-04-29

**Authors:** Yue Li, Qi Hu, Da-Wen Gao

**Affiliations:** ^1^State Key Laboratory of Urban Water Resource and Environment, Harbin Institute of Technology, Harbin 150090, China; ^2^School of Pharmaceutical Engineering, Shenyang Pharmaceutical University, Shenyang, Liaoning Province 110016, China

## Abstract

An integrated anaerobic fluidized-bed membrane bioreactor (IAFMBR) was investigated to treat synthetic high-strength benzothiazole wastewater (50 mg/L) at a hydraulic retention time (HRT) of 24, 18, and 12 h. The chemical oxygen demand (COD) removal efficiency (from 93.6% to 90.9%), the methane percentage (from 70.9% to 69.27%), and the methane yield (from 0.309 m^3^ CH_4_/kg·COD_removed_ to 0.316 m^3^ CH_4_/kg·COD_removed_) were not affected by decreasing HRTs. However, it had an adverse effect on membrane fouling (decreasing service period from 5.3 d to 3.2 d) and benzothiazole removal efficiency (reducing it from 97.5% to 82.3%). Three sludge samples that were collected on day 185, day 240, and day 297 were analyzed using an Illumina® MiSeq platform. It is striking that the dominant genus of archaea was always *Methanosaeta* despite of HRTs. The proportions of *Methanosaeta* were 80.6% (HRT 24), 91.9% (HRT 18), and 91.2% (HRT 12). The dominant bacterial genera were *Clostridium* in proportions of 23.9% (HRT 24), 16.4% (HRT 18), and 15.3% (HRT 12), respectively.

## 1. Introduction

The widespread use of antibiotics has generated large volumes of contaminated antibiotic wastewater. Antibiotics have not been degraded entirely even after passing through the processing of conventional wastewater treatment plants (WWTP) [1, 2]. They gradually enter the water environment when wastewater is discharged. Therefore, antibiotics have been detected in surface water [3, 4], groundwater, and soils, generating worldwide attention. The occurrence and release of antibiotics have adversely affected bioreactor treatment in decreasing COD removal efficiency because of their bacterial toxicity [5]. Furthermore, antibiotics are considered to be pollutants because antibiotics and their transformation products may lead to spread/transfer of antibiotic-resistant bacteria (ARB) and antibiotic resistance genes (ARGs) when microbes are exposed to antibiotics in the long term.

Among the processes used for wastewater treatment, anaerobic treatment has some technical advantages, such as the production of methane, lower energy costs, and lower excess sludge production [6]. Anaerobic bioreactors have been used for high-strength organic wastewater treatment, including treatment of contaminated antibiotic wastewater [5, 7]. However, anaerobic bioreactors alone cannot fulfill the demands of stringent effluent standards. To improve effluent quality, some researchers have combined anaerobic and membrane reactors [8–10]. A two-stage anaerobic fluidized-membrane bioreactor reportedly was used to treat municipal wastewater containing 20 pharmaceuticals, achieving pharmaceutical removal efficiencies of 78%–100% [11]. Also, membrane bioreactors could have an advantage in the release of antibiotic-resistant bacteria (genes). Munir et al. have researched the effluent and biosolids of five wastewater utilities in Michigan [12]. They found that membrane bioreactor has the least release of antibiotic-resistant bacteria (genes) compared to the four other types of wastewater treatment utilities.

Parameters such as hydraulic retention time (HRT), temperature, and solid retention time (SRT) have a significant effect on the performance and running life of a bioreactor. A large number of different combinations of operation conditions have been reported, such as SRT from a few days [13] to about a year [14], temperature from psychrophilic [15] to thermophilic, and HRT from a few hours [16] to a few days [17]. HRT is one of the essential operating conditions, which has a direct influence on the performance of the bioreactor [17]. In the light of different chemical compositions of antibiotic wastewater, it is important to select the corresponding HRT. The change of performance inevitably affects membrane fouling development in AnMBR. It has been reported that a decrease in HRT enhanced accumulation of soluble microbial products (SMP), which accelerated membrane fouling [18]. Our previous research showed the feasibility of an integrated anaerobic fluidized-bed membrane bioreactor treating synthetic benzothiazole wastewater [10]. However, little information is available about the influence of HRT on IAFMBR treating high-strength benzothiazole wastewater.

This study investigated the feasibility of an integrated anaerobic fluidized-bed membrane bioreactor (IAFMBR) to treat high-strength wastewater containing benzothiazole. This research was focused on the impact of hydraulic retention time (HRT) on the performance of the IAFMBR and the succession of microbial community structures.

## 2. Materials and Methods

### 2.1. Integrated Anaerobic Fluidized-Bed Membrane Bioreactor

**Figure 1:**
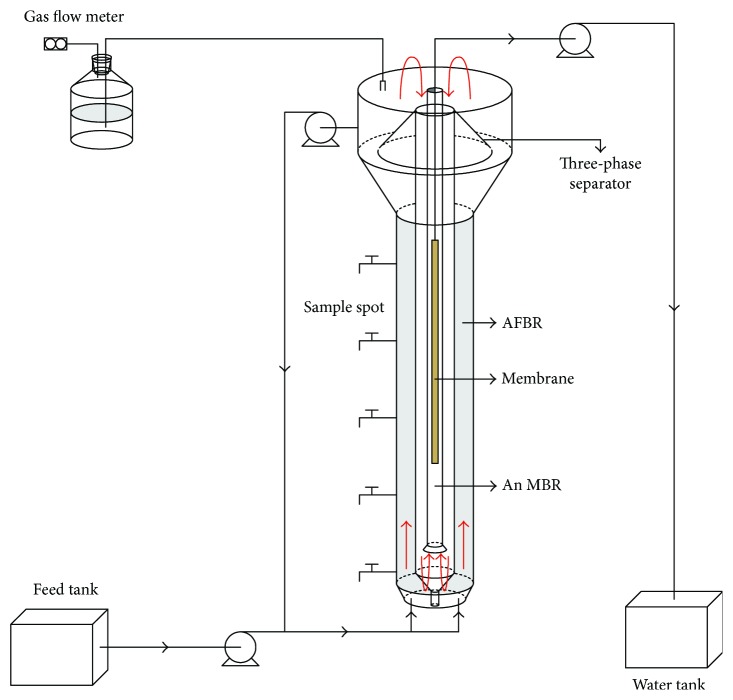
The schematic diagram of the IAFMBR.

The integrated anaerobic fluidized-bed membrane bioreactor (IAFMBR) was made of 10 mm Plexiglas with a total volume of 8.9 L (effective volume of 6.1 L) [10]. The reactor consisted of an outer tube, a middle tube, an inner tube, a three-phase separator, and a membrane module (Figure 1). The outer tube was filled with anaerobic granular sludge. A hollow fiber membrane (Mitsubishi Rayon Co., Ltd., Tokyo, Japan) was equipped in the inner zone with a total surface membrane area of 0.21 m^2^ and a pore diameter of 0.4 *μ*m. The designed membrane flux was 11.3 L/m^2^ h.

The IAFMBR consisted of an AFBR (anaerobic fluidized-bed reactor) and an AnMBR (anaerobic membrane bioreactor). The AFBR effluent was treated by anaerobic granular sludge. The IAFMBR effluent was a membrane permeate which was treated by anaerobic granular sludge and membrane.

### 2.2. Reactor Operation Conditions

The reactor had stably operated for 151 d, including a start-up phase (1–58 d) and an adaptation phase (59–151) (Table 1). In this study, the reactor was operated under HRT of 24 (152–185 d), 18 (186–240 d), and 12 h (241–297 d) (Table 1). Continuous membrane filtration was performed. During the whole experiment, the reactor was wrapped with an electrothermal wire to keep the temperature at 35°C. The SRT was 35 d, and the concentration of benzothiazole was 50 mg/L.

### 2.3. Inoculation and Feed Composition

The reactor was inoculated with 1.2 L anaerobic granular sludge that was taken from an anaerobic reactor treating wastewater from an alcohol-producing plant in Daqing, China. The MLVSS was 4850 mg/L, and the MLVSS/MLSS was 0.67.

Synthetic wastewater was fed according to the characteristics of antibiotic production wastewater coming from a pharmaceutical factory in Harbin, China. The concentration of benzothiazole was 50 mg/L. Glucose and acetate were used to maintain the COD (2961–3337 mg/L). The other compositions of wastewater were as follows (mg/L): NH_4_Cl, 140; urea, 40; KH_2_PO4, 45; MgSO_4_, 55; and CaCl_2_, 15. The inorganic nutrient composed is according to the previous study [8].

### 2.4. Sample Analysis

The COD was measured according to standard methods [19]. Biogas production was measured using a wet gas meter. Biogas production was detected using a gas chromatograph (Agilent GC 7890A, USA) with a thermal conductivity detector. The sample of VFAs was filtrated with a 0.45 *μ*m Millipore filter, and it was determined by a gas chromatograph (Agilent GC 7890, USA) equipped with a flame ionization detector. Benzothiazole concentration was detected by a high-performance liquid chromatography instrument (Waters e2695, USA) under ultraviolet detection set at 254 nm with a C18 column (SB-C18, 250 mm × 4.6 mm, Agilent Co., Ltd., USA). The mobile phase, flow rate, and temperature were as described previously[10].

The samples of mixed liquor were taken from the reactor. The sample of cake layer was taken from the membrane by flushing the membrane surface with a certain amount of deionized water. The extraction of EPS and SMP was based on [20]. Both SMP and EPS were quantified through a measurement of protein and polysaccharides. The concentration of proteins was detected by the modified BCA kit (Sangon Biotech Ltd., Shanghai, China) following the manufacturer's protocols. The concentration of polysaccharides was determined by the phenol sulphuric acid method [21].

### 2.5. Microbial Community Analysis

#### 2.5.1. Sample Collection and DNA Extraction

The sludge samples (HRT 24, 18, and 12 collected on days 185, 240, and 297, resp.) were taken from the AFBR reactor. Total DNA analysis was performed by extraction using a FastDNA SPIN Kit for Soil (MP Biomedicals (Shanghai) Ltd., China) following the manufacturer's protocols.

#### 2.5.2. PCR Amplification and Illumina® MiSeq Method

PCR amplification, production purification, and quantification were afforded by a sequencing company (Sangon Biotech Ltd., Shanghai, China). The extracted DNA was amplified using a set of bar-coded primers 341F and 805R for bacteria. The amplification of archaea DNA was used using nested PCR (two sets of primers). One set of primers was 340F and 1000R, and the other set of primers was 349F and 806R. The composition of different primers was reported in the previous study [10].

The thermocycling steps were as follows: 94°C for 3 min, followed by 5 cycles at 94°C for 30 s, 45°C for 20 s, and 65°C for 30 s; 20 cycles at 94°C for 20 s, 55°C for 20 s, and 72°C for 30 s; and a final extension step at 72°C for 5 min. The PCR productions were sequenced by an Illumina MiSeq high-throughput platform (Sangon Biotech Ltd., Shanghai, China).

#### 2.5.3. Biodiversity Analysis and Phylogenetic Classification

The raw reads were demultiplexed. The adapters, barcode, and primers in all reads were trimmed. Sequences shorter than 200 bp were removed with the PRINSEQ software. The UCHIME software was used to detect chimera sequences [10].

Operational taxonomic units (OTUs) were clustered by 97% similarity (3% dissimilarity level) using the UCLUST algorithm (http://www.drive5.com/uclust/downloads1_1-579.html). The Shannon index and Chao1 index were calculated to compare the diversity and richness of microbial structures [22].

## 3. Results and Discussion

### 3.1. Performance of IAFMBR

#### 3.1.1. COD Removal

**Figure 2:**
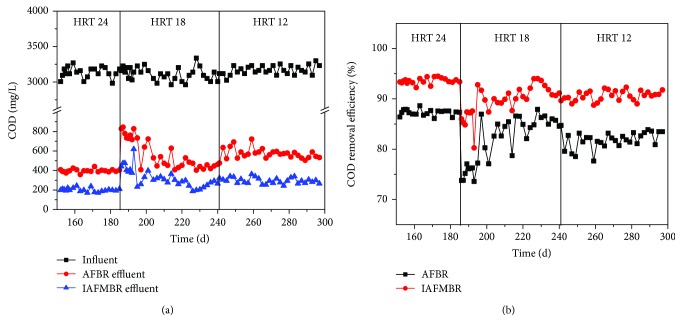
COD removal performance at different HRT. (a) Variations of COD concentration and (b) variations of COD removal efficiency.

The variations of COD were investigated during the three phases (Figure 2). In general, the COD removal efficiency of IAFMBR was relatively stable, and the numerical values were 93.6 ± 0.6%, 91.2 ± 1.7, and 90.9 ± 0.9% at the HRT of 24 h, 18 h, and 12 h. For AFBR, the COD removal efficiency was slightly impacted by the HRT. The COD removal efficiency was maintained at about 87.3 ± 0.6% at the HRT of 24 h, and the effluent COD was 398 mg/L. When the HRT was reduced to 18 h, the effluent COD was increased to 828 mg/L at the beginning and then decreased to 467 ± 57 mg/L at a stable period, corresponding to an efficiency of 84.9 ± 2.1%. However, as the HRT was reduced to 12 h, the effluent COD was 557 ± 28 mg/L, and the COD efficiency was 82.5 ± 1.1%.

The impact of HRT on the performance has been researched in some studies. The antibiotic wastewater that contained amoxicillin (AMX) was treated by an expanded granular sludge bed (EGSB) at an HRT of 8–20 h, and the COD removal efficiency dropped from 85% to 36.5% [23]. Gao et al. used IAFMBR treating domestic wastewater [8]. They found that the COD removal efficiency obviously decreased from 63.6 ± 2.5% (HRT 8) to 48.4 ± 2.6% (HRT 4). Compared to those studies that were previously mentioned, HRT variations did not obviously affect the COD removal efficiency in this study. This is because the synthetic feed (the main carbon sources were glucose and acetate) is easy to biodegrade by microorganisms.

#### 3.1.2. Benzothiazole Removal

**Figure 3:**
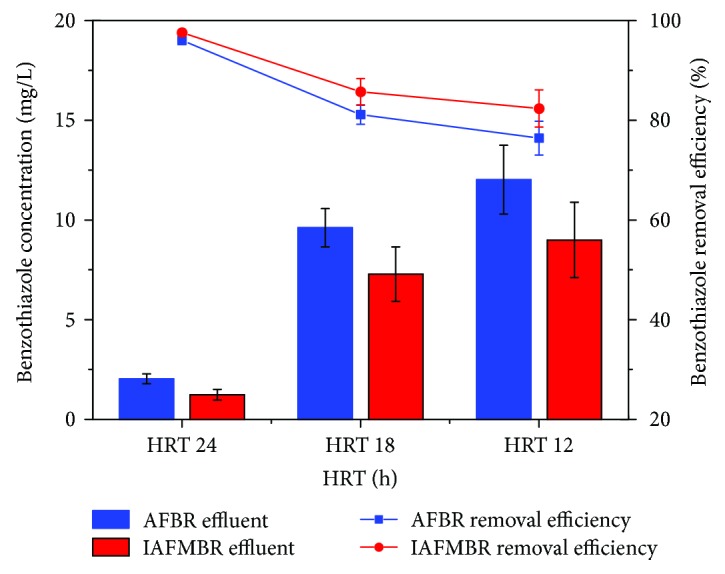
The variations of benzothiazole removal performance at different HRT.

Benzothiazole removal efficiency decreased with the stepwise drop of HRT (Figure 3). The average AFBR (IAFMBR) effluent benzothiazole concentrations were 2.03 ± 0.24 mg/L (1.23 ± 0.27 mg/L), 9.60 ± 1.36 mg/L (7.28 ± 1.36 mg/L), and 12.02 ± 1.71 mg/L (8.99 ± 1.89 mg/L) at the HRT of 24, 18, and 12 h. The benzothiazole removal efficiency of AFBR (IAFMBR) was 96.0 ± 0.5% (97.6 ± 0.5%), 81.1 ± 1.9% (85.7 ± 2.6%), and 76.4 ± 3.4% (82.3 ± 3.7%) at the HRT of 24, 18, and 12 h.

HRT is one of the critical factors that affect the degradation of antibiotics. It has been reported that the main removal pathway of benzothiazole was biodegradation [10]. For biodegradation, the contact time between biodegraded material and sludge was important which affects the treatment efficiency. For AFBR, a lower HRT applied may cause the washout of the functional bacteria that is required for the biodegradation of antibiotics [24]. For IAFMBR, the functional microbe could wash out and into the inner tube. However, the membrane fouling cycle was relatively short resulting in frequent membrane cleaning. The functional microbe could not enrich in the inner tube.

#### 3.1.3. VFA Accumulation

**Figure 4:**
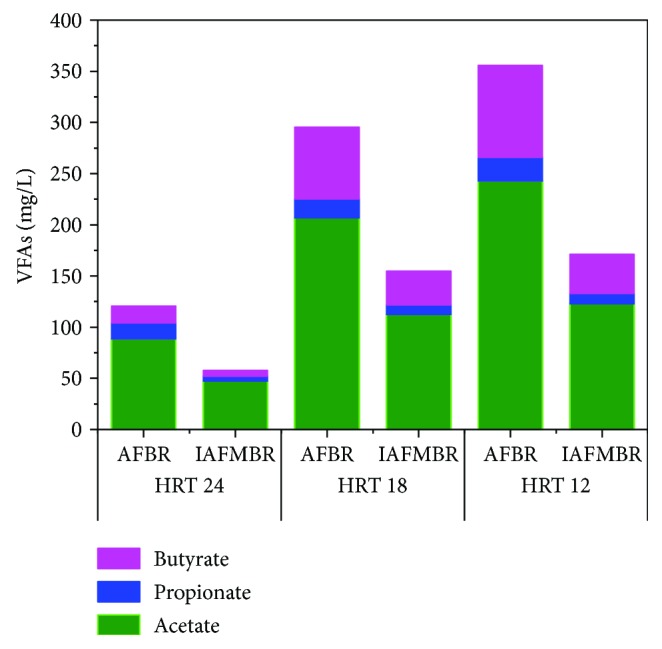
VFA accumulation during different HRT (average value) in AFBR and IAFMBR.

The accumulation and composition of the volatile fatty acids (VFAs) were supervised in different HRT (Figure 4). Acetate was the major component of VFAs in the AFBR effluent, which increased with the change of HRT, and its concentrations in the AFBR effluent were 88.44 ± 11.84 mg/L (HRT 24 h), 206.93 ± 15.58 mg/L (HRT 18 h), and 242.82 ± 9.55 mg/L (HRT 12 h), being accounted as about 73.31%, 69.98%, and 68.26% of total VFAS, respectively. The same phenomenon of acetate accumulation was also indicated in previous studies [8, 10]. Acetate is the substrate for acetotrophic methanogens which play an important role in CH_4_ production and for homoacetogenic bacteria, transforming acetate to hydrogen and CO_2_ [25].

The increment of propionate increased slightly, and its concentrations were 15.86 ± 3.31 mg/L, 18.84 ± 5.75 mg/L, and 23.01 ± 0.79 mg/L at the HRT of 24, 18, and 12 h in the AFBR effluent. The concentration of butyrate increased from 16.33 ± 3.07 mg/L (HRT 24 h) to 69.93 ± 9.10 mg/L (HRT 18 h) to 89.91 ± 4.14 mg/L (HRT 12 h) in the AFBR effluent, being accounted as about 13.54%, 23.65%, and 25.27% of total VFAs, respectively. It was reported that antibiotics had an adverse effect on butyrate-oxidizing bacteria [25]. In this study, the residual concentration of benzothiazole increased with a decreased HRT (Figure 3), which could inhibit butyrate degradation. A similar inhibition of butyrate degradation was found in other antibiotics [26]. Valerate was not detected during all periods.

In general, tVFA accumulation increased with the declining HRT. The tVFAs in IAFMBR effluent was lower than those in AFBR, which were 57.83 mg/L ± 13.81 mg/L, 154.66 ± 18.50 mg/L, and 171.04 ± 10.88 mg/L at the HRT of 24 h, 18 h, and 12 h.

#### 3.1.4. Biogas Production

Biogas production was monitored throughout the three phases of reactor operation (Table 2), particularly for the evaluation of methanogenic activity. The biogas production volume was greatest at HRT of 12 h (21.49 ± 0.39 L/d) compared with that of HRT of 18 (14.00 ± 0.78 L/d) and 24 h (10.74 ± 0.39 L/d). Methane production apparently increased from 7.60 ± 0.26 L/d to 10.29 ± 0.57 L/d to 14.88 ± 1.57 L/d at HRT of 24, 18, and 12 h. These data showed that methane production augmented with an increase in the organic loading rate, which was similar to previous studies [8].

However, the methane percentage was slightly affected by the change of HRT (70.9 ± 0.3%, 73.5 ± 2.1%, and 69.3 ± 1.6% at HRT of 24, 18, and 12 h). About 70% methane content was similar to previous studies [25]. The methane yield is a useful parameter to evaluate the performance of an anaerobic reactor [5]. The methane yield was relatively stable, and the values were 0.309 ± 0.014 m^3^ CH_4_/kg COD_removed_ (HRT24), 0.327 ± 0.028 m^3^ CH_4_/kg COD_removed_ (HRT18), and 0.316 ± 0.022 m^3^ CH_4_/kg COD_removed_ (HRT12), respectively. There are two possible reasons. On the one hand, methanogens were in the anaerobic granular sludge. This structure protected the activity of methanogens. On the other hand, the effect of BTH on the methanogens was not significant.

**Figure 5:**
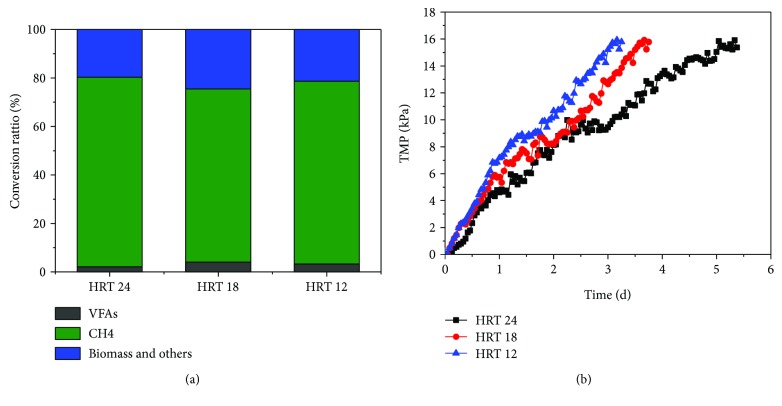
Mass balance and transmembrane pressure (TMP). (a) Mass balance at different HRT and (b) TMP profile at different HRT.

In order to show the carbon flow, a mass balance (based on COD) was illustrated (Figure 5(a)). About 70% carbons were converted to methane at different HRT. The data of mass balance and methane yield (Table 2) was similar, which showed that the production of methane was not affected by the reducing HRT.

### 3.2. Membrane Fouling

#### 3.2.1. TMP Fraction

The change of transmembrane pressure (TMP) was used as an indicator of membrane fouling. Clean-up or backflushing was not applied in order to detect the one-time operational duration of membrane fouling. In this experiment, the TMP was collected at the stable period of different HRTs, and the value of TMP reached 16 kPa as membrane fouling.

In general, the trends of the membrane fouling cycle were similar at different HRT (Figure 5(b)). The membrane fouling cycle was 5.3, 3.7, and 3.2 d at HRT of 24, 18, and 12 h. When HRT was 24 h, the TMP rapidly increased to 9 kPa on day 2.4 in a linear manner and then had transient platform fluctuations. Finally, TMP was close to 16 kPa on day 5.3 in a linear manner again.

Generally, with the shortened HRT, the influent COD of AnMBR was increased (from 398 mg/L to 557 mg/L) (Figure 2(a)), which led to the decrease in the membrane fouling cycle. These results were similar to previous studies [8]. Gao et al. have researched the control of membrane fouling by addition of granular-activated carbon (GAC) at HRT 4, 6, and 8 h in an anaerobic membrane bioreactor. They found that the membrane fouling cycle at HRT 4 h (about 15 d) was almost two times of that at HRT 8 h (about 31 d) when 40 g GAC was added. The membrane fouling cycle of this study was obviously short. The possible reasons are as follows: (1) no addition of GAC. The fluidization of GAC could evidently reduce TMP [16]. However, maintenance of the fluidization of GAC demands a lot of energy to consume. (2) The feed had high COD (2961–3337 mg/L) and antibiotic (50 mg/L benzothiazole), which resulted in aggravation membrane fouling.

#### 3.2.2. EPS and SMP Fraction

**Figure 6:**
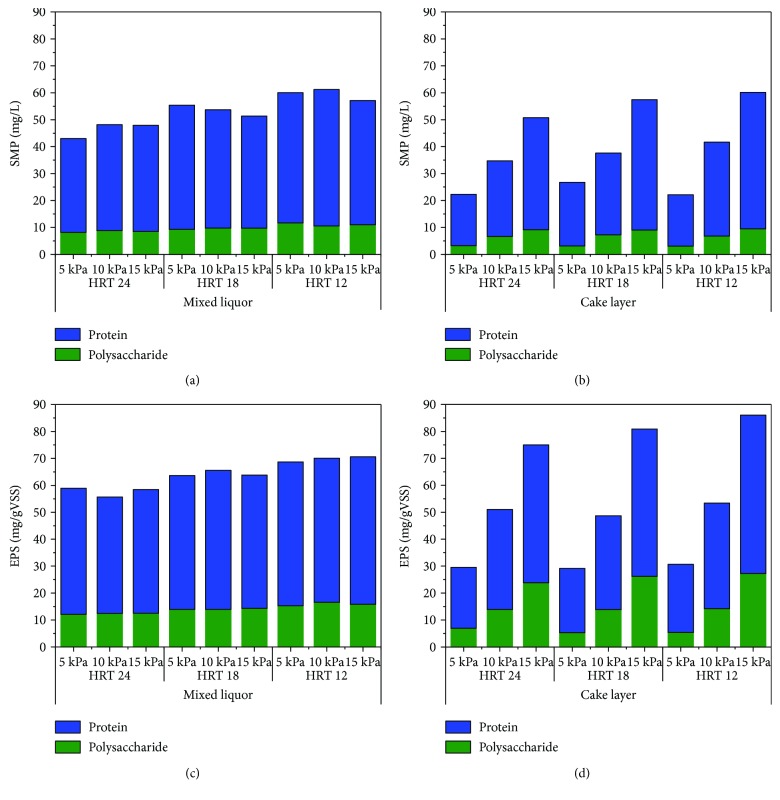
The variation of extracellular polymeric substances (EPS) and soluble microbial products (SMP) in the mixed liquor and cake layer. (a) The SMP in the mixed liquor, (b) the SMP in the cake layer, (c) the EPS in the mixed liquor, and (d) the EPS in the cake layer.

The variations of EPS and SMP, from both mixed liquor and cake layer, in different TMPs were detected (Figure 6). In the mixed liquor, there was no significant difference in SMP under different HRT. For instance, when the HRT was 24 h, the SMP were 43.00 mg/L, 48.19 mg/L, and 47.88 mg/L at TMP of 5 kPa, 10 kPa, and 15 kPa, respectively (Figure 6(a)). The concentrations of EPS and SMP were different at HRT 24, 18, and 12 h in the mixed liquor, but the trends were similar. EPS and SMP were not affected by TMP variations in each certain HRT with stable performance (Figures 6(a) and 6(c)). The possible reason was that the mixed liquor and microbiology communities were relatively stable, which did not change with TMP.

However, for cake layer, the concentrations of EPS and SMP increased with rising TMP in each certain HRT (Figures 6(b) and 6(d)). For instance, when the HRT was 24 h, the SMP were 22.28 mg/L, 34.74 mg/L, and 50.73 mg/L at TMP of 5 kPa, 10 kPa, and 15 kPa, respectively. Those EPS and SMP in the cake layer came from the biomass growth with rising TMP on the membrane surface. Sludge cake formation on the membrane surface is viewed as the major cause of membrane fouling [27]. It has been reported that cake sludge deposited on the membrane surface has much higher specific filtration resistance than that of bulk sludge liquor [28].

The concentrations of EPS and SMP in mixed liquor and the cake layer increased with decreasing HRT, which was due first to the faster growth of anaerobic sludge with shorter HRT [18]; secondly, more undegraded substrates were present in the mixed liquor. It has been reported that SMP occurs in response to environmental stress, such as that caused by toxic compounds [29]. In this study, the concentration of benzothiazole was increased (from 1.23 ± 0.27 mg/L to 12.02 ± 1.71 mg/L) with the change of HRT, which could explain the increasing SMP. The major fraction of SMP was the soluble phase of EPS, and SMP consistently varied with EPS in the aerobic MBR [30].

The concentration of protein was much higher than that of polysaccharide either in mixed liquor or cake layer, in EPS or SMP, which was consistent with previous studies[8]. Meng et al. found that proteins are more hydrophobic, adhere more easily to the membrane surface, and induce membrane fouling [31]. In addition, our group has reported that protein had a negative impact on membrane fouling compared to polysaccharide [32]. This conclusion explained the cause of serious membrane fouling in another aspect.

### 3.3. Microbial Community Structure

Normally, bacteria play a dominant role in antibiotic wastewater treatment systems: bacteria carbon transformation functions may be disturbed. Meanwhile, bacteria possessing antibiotic resistance could survive in this condition [33]. That is why it is important to understand the microbial community structure.

#### 3.3.1. Bacterial Community Analysis

The Illumina MiSeq high-throughput platform was used to determine three microbial samples (HRT 24, 18, and 12 collected on days 185, 240, and 297, resp.), which were taken from the AFBR. The qualified sequencing reads were in the range of 24,182 to 42,241, which were clustered in more than 1500 OTUs based on a threshold of 97%.

**Figure 7:**
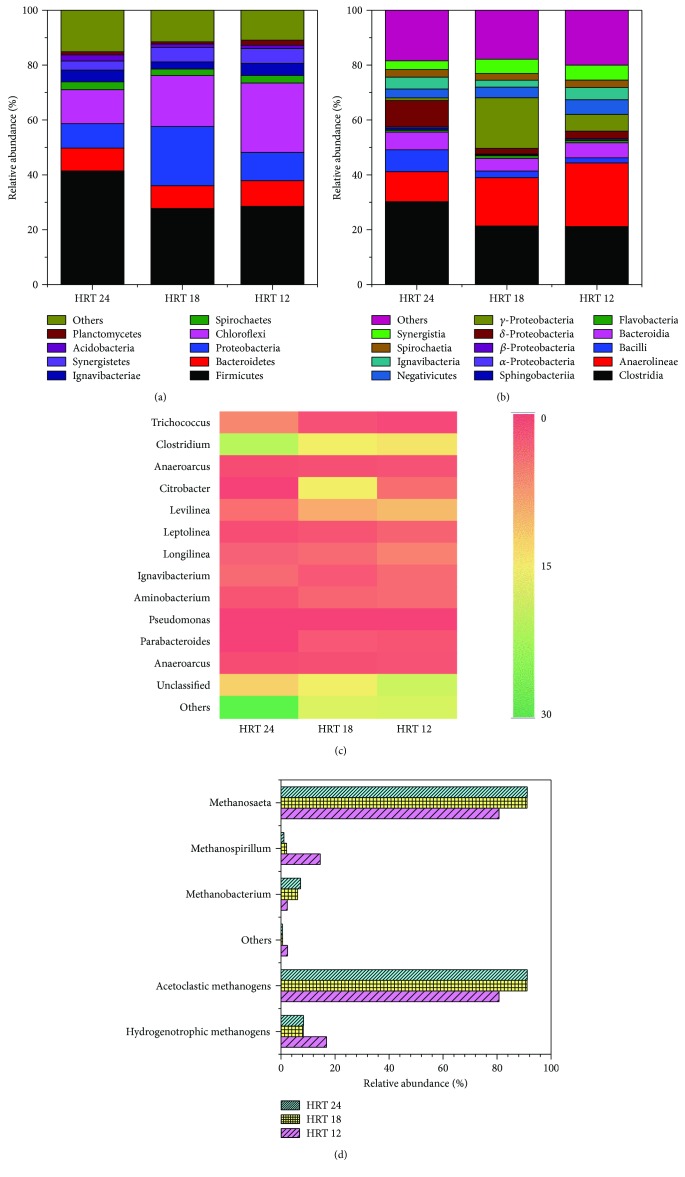
Taxonomic classification of bacteria and archaea form different HRT (collected on day 185, 240, and 297): (a) bacteria phylum, (b) bacteria class, (c) bacteria genus, and (d) archaea genus.

The relative abundance of phylum, class, and genus levels was described in order to understand the communities better. More than 20 types of bacterial phyla were recovered altogether, and the main phyla were Firmicutes (27.7%–41.4%), Proteobacteria (8.9%–21.6%), Chloroflexi (12.4%–25.3%), and Bacteroidetes (8.3%–9.4%) (Figure 7(a)). These phyla were found to be significant microbial groups in other anaerobic bioreactors treating antibiotic wastewater [7, 9, 10].

At the class level, two of the most important classes were Clostridia (21.2%–30.2%) and Anaerolineae (10.9%–23.2%), (Figure 7(b)). The sample of HRT 24 was dominated by Clostridia (30.2%), Anaerolineae (10.9%), and *δ*-Proteobacteria (9.6%). The HRT 18 community was dominated by Clostridia (30.2%), followed by *γ*-Proteobacteria (18.4%) and Anaerolineae (17.6%). The HRT 12 community was dominated by Anaerolineae (23.2%) and Clostridia (21.2%). Clostridia had many carbon-degrading functions, which played a main role in COD removal. Some Clostridia were able to cleave aromatic rings and utilize the methyl group of aromatic methyl ethers as carbon source [34, 35]. Clostridia and *δ*-Proteobacteria were the major classes associated with antibiotic environments [33, 36]. Moreover, Anaerolineae was found in an anaerobic bioreactor [37].

In general, microbes from two samples showed similar diversities but different abundance. The sample of HRT 24 was dominant by *Clostridium* (23.9%), followed by *Trichococcus* (6.9%), and *Levilinea* (4.8%). The major community in a sample of HRT 18 was *Clostridium* (16.4%), followed by *Citrobacter* (16.3%) and *Levilinea* (10.0%). The sample of HRT 18 was dominant by *Clostridium*, *Levilinea*, and *Longilinea* in the proportion of 15.3%, 11.6%, and 6.3% (Figure 7(c)).

In this study, no matter how the condition changes, *Clostridium* was the dominant genus, which was a common genus of dominant bacteria in anaerobic bioreactors [38, 39]. *Clostridium* belonging to phyla of Firmicutes with hard cell walls can produce endospores. *Clostridium* spp. were reported to have the ability of degrading complex organic matters from acid by producing or secreting hydrolases, such as protease and *α*-amylase[40]. The relative abundance of some genera was increased in response to HRT, such as *Levilinea*, *Leptolinea*, and *Longilinea*. *Levilinea*, *Leptolinea*, and *Longilinea* are Gram-negative, belonging to the class of Anaerolineae and phyla of Chloroflexi with flexible filaments [41, 42]. Meanwhile, the decreasing HRT led to the increasing residual concentration of benzothiazole. This phenomenon indicated that the three genera could be inclined to develop in residual benzothiazole.

#### 3.3.2. Archaeal Community Analysis

Over 30,000 qualified sequences were produced by an Illumina MiSeq high-throughput platform. The dominant genus of the archaeal community was *Methanosaeta* in proportions of 80.8% (HRT 24), 91.1% (HRT 18), and 91.2% (HRT 12) followed by *Methanospirillum* (14.5%, 2.1%, and 1.1% in HRT 24, 18, and 12, resp.) and *Methanobacterium* (2.3%, 6.1%, and 7.3% in HRT 24, 18, and 12, resp.) (Figure 7(d)). The proportion of acetotrophic methanogens (*Methanosaeta*) increased from 80.8% to 91.2%, and the proportion of hydrogenotrophic methanogens (*Methanospirillum* and *Methanobacterium*) decreased from 16.9% to 8.3%. Overall, the dominant participant was always *Methanosaeta* (acetotrophic methanogens), no matter how the HRT changes.

*Methanosaeta* was an important archaea in anaerobic bioreactors [43, 44]. *Methanosaeta* belongs to acetotrophic methanogens which can convert acetic acid to methane and CO_2_, and this process produces 70% of methane [45]. Wang et al. treated brewery wastewater using a continuous stirred microbial electrochemical reactor (CSMER) [37]. The CSMER comprised a complete mixing zone (CMZ) and microbial electrochemical zone (MEZ), and the anaerobic sludge was inoculated in CMZ. They found that *Methanosaeta* (40.3%) was the predominant archaea in CSMER_CMZ_ and *Methanosaeta* existed in each sample. *Methanosaeta* have been found to have high methane yield so that the higher relative abundance of *Methanosaeta* manifested a favorable condition for methane yield [46]. And this finding was in line with the higher methane yield in HRT 18 (0.327 m^3^ CH_4_/kg COD_removal_) and 12 (0.327 m^3^ CH_4_/kg COD_removal_) compared with HRT 24 (0.315 m^3^ CH_4_/kg COD_removal_).

## 4. Conclusions

This study indicated the feasibility of an IAFMBR to the treatment of high concentration wastewater containing antibiotics at different HRT. The COD removal efficiency, the methane percentage, and the methane yield were not affected by HRT decreasing from 24 h to 12 h. The decreased HRT had an adverse effect on membrane fouling and benzothiazole removal efficiency. For bacteria, the dominant phyla, class, and genera were Firmicutes, Clostridia, and Clostridium. For archaea, the dominant genera were *Methanosaeta.* With the decreased HRT, the acetotrophic methanogens increased while that of hydrogenotrophic methanogens decreased. The best performance was obtained at HRT of 24 h.

## Figures and Tables

**Table 1:** The summary of operating conditions of IAFMBR system.

Phase	Start-up	Adaptation	HRT 24	HRT 18	HRT 12
Days (d)	1–58 d (58 d)	59–151 d (93 d)	152–185 d (34 d)	186–240 d (55 d)	241–297 d (57)
Benzothiazole (mg/L)	0	1–50	50	50	50
HRT (h)	24	24	24	18	12
Temperature (°C)	35	35	35	35	35
OLR (kg COD/m^3^·d)	3.33	3.26	3.13	4.64	6.36

**Table 2:** The biogas production at different HRTs (average concentrations at steady-states).

HRT	Biogas production (L/d)	Methane production (L/d)	Methane percentage (%)	Methane yield (m^3^ CH_4_/kg·COD_removed_)
24	10.74 ± 0.39	7.60 ± 0.26	70.9 ± 0.3	0.309 ± 0.014
18	14.00 ± 0.78	10.29 ± 0.57	73.5 ± 2.1	0.327 ± 0.028
12	21.49 ± 1.26	14.88 ± 1.57	69.3 ± 1.6	0.316 ± 0.022
